# Immunohistochemistry as an Important Tool in Biomarkers Detection and Clinical Practice

**DOI:** 10.4137/bmi.s2185

**Published:** 2010-02-09

**Authors:** Leandro Luongo de Matos, Damila Cristina Trufelli, Maria Graciela Luongo de Matos, Maria Aparecida da Silva Pinhal

**Affiliations:** Biochemistry Department, Faculdade de Medicina do ABC, Santo André, SP, Brazil. Email: lmatos@amcham.com.br

**Keywords:** immunohistochemistry, review, pathology

## Abstract

The immunohistochemistry technique is used in the search for cell or tissue antigens that range from amino acids and proteins to infectious agents and specific cellular populations. The technique comprises two phases: (1) slides preparation and stages involved for the reaction; (2) interpretation and quantification of the obtained expression. Immunohistochemistry is an important tool for scientific research and also a complementary technique for the elucidation of differential diagnoses which are not determinable by conventional analysis with hematoxylin and eosin. In the last couple of decades there has been an exponential increase in publications on immunohistochemistry and immunocytochemistry techniques. This review covers the immunohistochemistry technique; its history, applications, importance, limitations, difficulties, problems and some aspects related to results interpretation and quantification. Future developments on the immunohistochemistry technique and its expression quantification should not be disseminated in two languages—that of the pathologist and another of clinician or surgeon. The scientific, diagnostic and prognostic applications of this methodology must be explored in a bid to benefit of patient. In order to achieve this goal a collaboration and pooling of knowledge from both of these valuable medical areas is vital

## The Immunohistochemistry Technique

The immunohistochemistry technique is used in the search for cell or tissue antigens ranging from amino acids and proteins to infectious agents and specific cellular populations.^1^ The technique comprises two phases: (1) slide preparation (specimen fixation and tissue processing) and stages evolved for the reaction (in order: antigen retrieval, non-specific site block, endogenous peroxidase block, primary antibody incubation, and the employment of systems of detection, revealing and counterstaining and also slide mounting and storage); (2) interpretation and quantification of the obtained expression.^2^

Immunohistochemistry is an umbrella term that encompasses many methods used to determine tissue constituents (the antigens) with the employment of specific antibodies that can be visualized through staining.^1^,^3^ When used in cell preparations it is called immunocytochemistry, a term that some authors use for all methods entailing the immunological search of cell antigens, even when this involves tissue slices.

Brandtzaeg stated that immunostaining for cell markers represents a way to “talk with cells”, because it allows not only the histological origin of the cell to be identified but also indicates its function *in vivo*, when duly investigated with the correct antibodies.^1^

The same author affirmed that it is lamentable and non-justifiable to classify immunohistochemistry as a merely descriptive method. He also emphasized that many reviewers, not aware of the accuracy of immunological detection methods, may consider them an inferior research tool where many manuscripts are refused on these grounds. He concluded that *in vitro* and *in situ* trials are in fact “pictures” of the situations that occur *in vivo* and therefore constitute one of the pillars of biomedical research. This includes immunohistochemistry, the importance of which is growing.^1^

### History

The history of immunostaining methods began when Marrack produced reagents against typhus and cholera microorganisms, using a red stain conjugated to benzidin tetraedro.^4^ However, Professor Albert H. Coons from Harvard School of Medicine—Boston, U.S.A. believed that the antigen detection provided by red color in tissue slices had very low sensitivity under optical microscopy and, in the early nineteen forties demonstrated that localizing antigens, especially microorganisms, was possible in tissue slices using antibodies against *Streptococcus pneumoniae* stained with fluorescein, visualized by ultra-violet light (fluorescence microscopy).^5^

Subsequently, the introduction of enzymes as marked antibodies, developed by Nakane, heralded a new and important era for immunohistochemistry, since it was possible to see these reactions through optical microscopy. These results had great impact and were much awaited in the nineteen sixties.^6^–^8^ This innovation took immunohistochemistry beyond the exclusive sphere of laboratories equipped with fluorescence microscopes, and the technique spread to a broad group of researchers and pathologists.^3^

The following discoveries of the unlabelled antibody peroxidase-antiperoxidase (PAP) method by Sternberger et al^9^ and the alkaline phosphatase-antialkaline phosphatase (APAAP) method by Mason et al^10^,^11^ significantly expanded the application of immunohistochemistry technique.^9^,^12^ The diaminobenzidine molecule (DAB) was also conjugated to antibodies during the same period,^13^ currently representing the most used chromogen for peroxidase, and as it produces an electrodense precipitate which is also used in electronic microscopy, substituting ferritin.^14^ Subsequently, gold colloidal particles were introduced as immunohistochemical colorations^15^ and this finding rapidly led to an important method of subcellular immunostaining.^16^

The discovery of antigen retrieval methods (exposure of antigen epitopes present in study tissue, favoring the antigen-antibody reactions for the next stages of the technique) by Huang et al,^17^ and also the systems of secondary antibody detection (for example the avidin-biotin-peroxidase complex—ABC and the labeled streptavidin-biotin complex—LSAB) by Hsu et al^18^–^21^ allowed immunohistochemistry to be used in fresh specimens as well as in fixed tissues, which further increased the applicability of the technique in pathology diagnostic routines. However, only after the presence of tissue antigens could be demonstrated by the immunoperoxidase technique in tissues fixed in formalin and embedded in paraffin, did immunohistochemistry really became incorporated into the diagnostic routine of pathological anatomy.^22^–^27^

**Figure 1.**
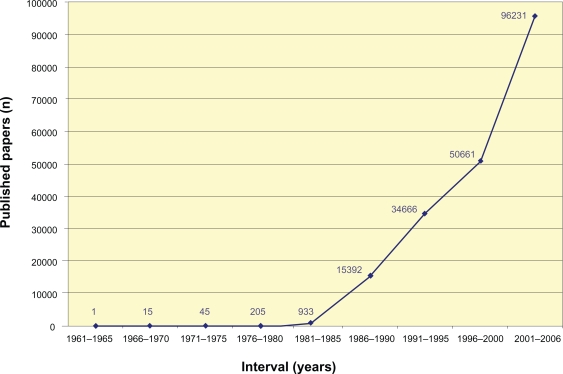
Number of scientific publications using the immunohistochemistry technique, found on the Medline database, between 1960 and 2006. The plot indicates the frequency in which the term “immunohistochemistry” appears in the title or abstract of the manuscripts. (Adapted from Werner et al^27^).

In the last couple of decades there have been an exponential increase in publications on immunohistochemistry and immunocytochemistry techniques (Fig. 1). This literature is available in many cellular and molecular biology, biochemistry, pathology, histology, immunology, internal medicine and surgery scientific articles.

This fact reflects the position that immunohistochemistry currently holds in a pathological anatomy laboratory. It is an important tool for scientific research and also a complementary technique in the elucidation of differential diagnosis which are not determinable by conventional analysis with hematoxylin and eosin.^2^,^22^–^24^,^26^,^28^–^37^ The great improvement in the contribution and application of immunohistochemistry in pathological anatomy became known as the “brown revolution” of the histopathology laboratory.^22^

### Applications and importance

**Figure 2.**
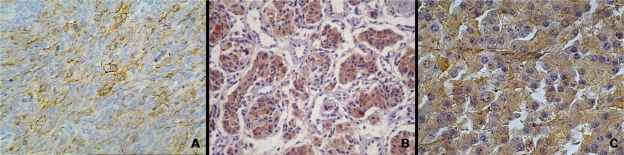
Histogenetic diagnosis of neoplasias using immunohistochemistry technique. **A**) Expression of cytoqueratin AE1/AE3 in lung carcinosarcoma (IHC-peroxidase—X200); **B**) chromogranin expression in gastric neuroendocrine carcinoma (IHC-peroxidase—X100); **C**) HMB 45 immunostainning in murine melanoma (IHC-peroxidase—X400).

**Figure 3.**
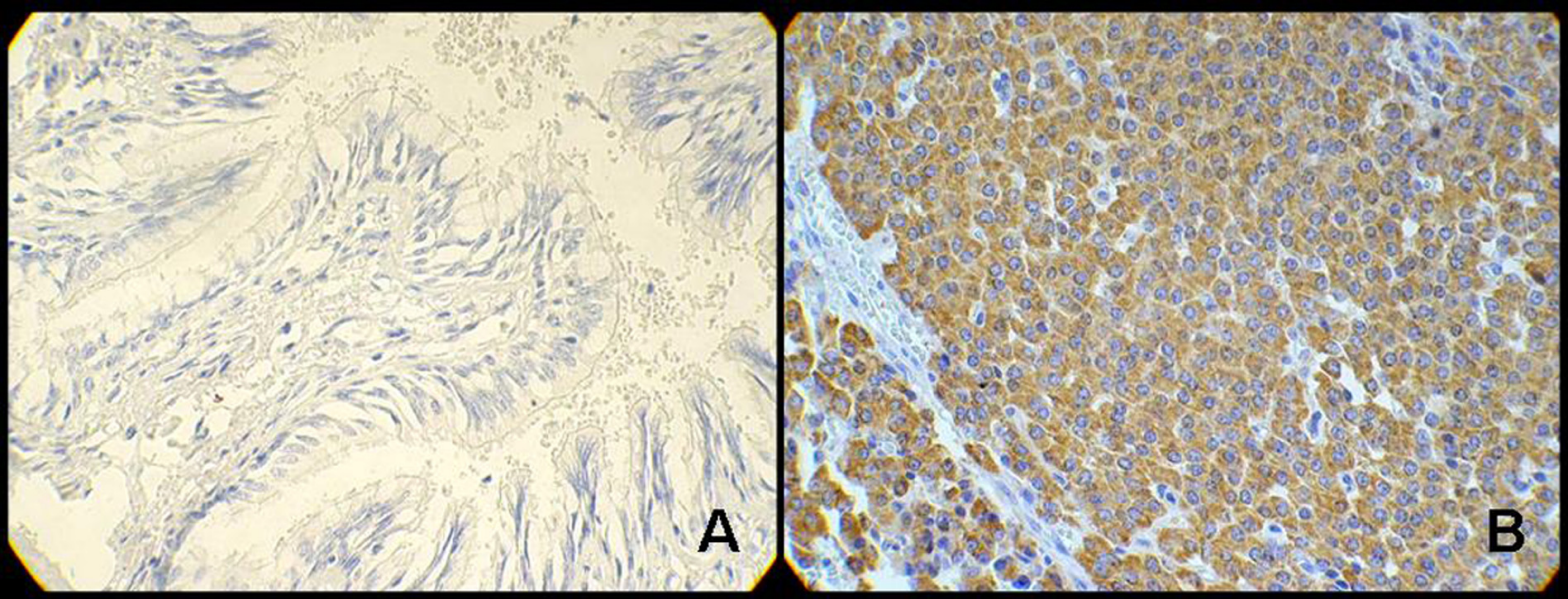
Heparanase expression in the diagnosis of broncopulmonar carcinoid tumors. Optical microscopy at X400 power: **A**) negative expression of heparanase (absence of staining—peroxidase—in cell’s cytoplasm) in bronchial mucosa not compromised by neoplasm; **B**) positive expression of heparanase (presence of cytoplasm full of peroxidase—brownish areas) in broncopulmonar carcinoid tumor. (Adapted from: de Matos et al^87^).

The immunohistochemical reactions can be used in different situations within research or pathological anatomy laboratories. The most important are: 1) histogenetic diagnosis of morphologically non-differentiated neoplasias (Fig. 2); 2) subtyping of neoplasias (such as lymphomas, for example); 3) characterization of primary site of malignant neoplasias; 4) research for prognostic factors and therapeutic indications of some diseases; 5) discrimination of benign *versus* the malign nature of certain cell proliferations (Fig. 3); identification of structures, organisms and materials secreted by cells.^2^,^22^,^23^,^26^,^36^,^38^

Werner and colleagues^27^evaluated the reason for employment and number of cases in which immunohistochemistry aided the diagnosis of neoplasias and pseudo-neoplastic lesions. It was noted that from a total of 3,760 specimens, in 19% of cases immunohistochemistry was used to determine prognostic factors or proliferative indexes; 17% of cases had the purpose of identifying microorganisms, cells, structures or secreted materials; and 64% of cases had a diagnostic application. From the 835 cases of this latter category immunohistochemistry contributed in 83% for specific diagnoses and decreased the number of non-defined diagnosis by 12%. In 5% of cases immunohistochemistry did not aid the pathologist due to the exiguity of some samples, presence of extensive necrosis, or extreme non-differentiation of some neoplasias. Data present in the literature on this subject is rare, however this study corroborates the results shown.^22^,^30^,^32^ They therefore concluded that immunohistochemistry is a helpful complementary diagnostic method in 95% of cases and contributes toward surgical and therapeutic conducts, with low cost and high benefit.^27^

### Limitations, difficulties and problems

Although a relatively simple technique, immunohistochemistry has some particularities and its outcome depends on many factors. The usefulness and contribution of immunohistochemistry in solving problems in pathological anatomy is directly proportionate to the experience of the hands that perform the reactions and also the eyes that interpret the results.^2^,^22^,^24^,^36^,^38^ Therefore, even though very simple in concept, immunostaining methods requires rigor of execution and may present significant bias. Hence, its outcomes must be interpreted with caution.

A recent review^39^ discusses the main bias that may follow the analysis of immunohistochemistry reactions. These are didactically divided into reaction bias (examples: specimen fixation, tissue processing, antigen retrieval and detection system) and interpretation bias (examples: selection of antibody panels, sensitivity of the chosen panel, choice of antibody types and clones, results and literature interpretation).

A wide variety of protocols for standardizing the immunohistochemistry technique are being proposed to minimize undesirable effects. The Committee of Quality Control in Immunohistochemistry of the French Pathology Society published a report in 1997 demonstrating that two of the main causes of diagnosis mistakes in immunohistochemistry are the non-employment of antigen retrieval techniques and the use of amplifying methods with low power. Other renowned international quality programs are the electronic database *Immunoquery* (“*Immunohistochemistry Literature Database Query System*”) and the UK NEQAS quality program (*“United Kingdom National External Quality Assessment Scheme for Immunocytochemistry”*).^40^–^42^

The acquisition, handling, fixation, specimen delivery to the laboratory and antigen retrieval are all critical factors. Fresh specimens that are inadvertently submitted to long periods of fixation may significant lose antigenicity.^39^,^43^ As an example, Jacobs and colleagues^44^ showed that there is progressive loss of antigenicity upon only 12 week storage of breast cancer histological slices on slides stored in ambient temperature for the detection of p53, Bcl-2, estrogen receptor and factor VIII proteins. However, the same was not observed in recent histological slices of specimens in paraffin blocks for periods of over 10 years.^45^ The specimen fixation in formaldehyde and its consequent inclusion in paraffin are the internationally most used histological processing procedures. Some specialists propose that this procedure should be the standard for comparing diagnostic outcomes among immunohistochemistry reactions.^46^ However, formaldehyde fixation results in a variably reversible loss of immunoreactivity by its masking or damaging some antibody binding sites.^29^ Although such epitopes may be demasked by several epitope retrieval methods, the immunohistochemical detection system must still be sensitive enough to produce a strong signal. For some epitopes, the duration of the formaldehyde fixation is critical. With some antibodies, depending on the resistance of its target epitope to autolytic change, delay in fixation may cause loss of immunoreactivity.^47^

Other fixatives often used in pathology include alcohol and alcohol-based fixatives such as acetone. Alves et al^48^ studied the fixation in ethanol and formalin for trypsin digestion in immunohistochemical detection of cytokeratins and vimentin in a case of ovarian cystadenofibrocarcinoma. They found superior reactivity for both markers in achieved ethanol-fixed sections, even in samples stocked up to 60 days. Cytokeratin reaction in formalin-fixed sections was better when trypsin was used. However, this digestion was deleterious to vimentin detection. This was an import work to alert surgeons and oncologists on the relevance of fixation of specimens suspicious for neoplasia, since different epitopes may require different fixatives and the inadequate choice in the operative room may impart difficulties when immunohistochemistry is necessary.

It is important to emphasize that in tissue processing, inclusion in paraffin at high temperatures (in general, over 60 °C) may compromise the specimen antigenicity. Another important point addresses the preparation of slides. The block slices must preferentially present a thickness ranging between 3 and 7 μm and must be deposited on slides previously prepared with some kind of adhesive (the most used are silane and polylysine). Slices less than 3 μm thick could result in very weak immunostaining while those thicker than 7 μm may lead to loss of tissue on the glass slide or may hamper analysis of the resultant immunostaining.^39^

The amount of material to be analyzed is being discussed, especially now that pathologists are expected to reach a precise diagnosis with small samples.^2^ In the majority of situations a block is sufficient, preferentially when it contains a fragment of the tumor-surrounding parenchyma interface (prepared in the macroscopic examination), distally to hemorrhagic or extensively necrotic areas, as well as a fragment representative of the tissue distal to the neoplasm.^49^ Whenever possible, tissue that was previously submitted for frozen examination must be avoided.^2^

Regarding antigen retrieval, the simplification of procedures, costs and technical error risk reduction are important factors. Irradiation techniques with microwaves or by humid heat in pressure or vapor pan, with exposition times adapted to offer the same pattern of staining in a group of case-controls has been suggested.^2^,^50^,^51^

The use of detection systems (secondary antibodies) is also considered valuable in error reduction.^52^ Among high discharge amplification systems, the avidin-biotin-peroxidase complex (ABC) and the labeled streptavidin-biotin complex (LSAB) are the most important.^53^–^56^ Specific situations require adaptations and even the use of alternative detection methods.

The selection of an adequate method is one of the great technical responsibilities faced in an immunohistochemistry laboratory. The advance in the technique, with systems of epitope retrieval through heat (HIER) and amplification methods, as well as the reactions performed in a single stage (EPOS)^57^ and the method of catalyzed product deposition (CARD),^58^,^59^ have introduced a paradox in immunohistochemistry. On the one hand numerous cases hitherto unsolved because of negativity in many panels, became positive and began to permit precise diagnosis. On the other hand, antibodies that were expressed characteristically in certain neoplasias began to react non-specifically in other situations.^2^,^25^ Concerned about the so called “anarchy” then introduced, Swanson^25^ proposed that no method should be universally applicable, the choice should be based on the technique that, in the experience of the laboratory or of the school followed by researchers, best solves the diagnostic question.^2^

Due to their flexibility and relatively low cost, the most used protocols currently (such as the ABC method, for example) are indirect and therefore require many stages of incubation. High sensitivity could be obtained with the application of immunological principles, enzymatic amplification reactions and/or the employment of avidin-biotin complex, however the various steps required must be rigorously followed in order to avoid non-desirable interactions. It is fundamental that, on technical planning, all reagents follow the sequence rigorously established, where the employment of work flow charts for such stages are very useful in avoiding false results. Making notes of all reaction stages and pattern of each antibody are equally important and are suggested in patterning technique programs.^2^

The ability of the specialized technician who performs the reactions is a guarantee against the introduction of crossed immunological reactions with endogenous immunoglobulins during the test preparations, or with different sequence experiments of immunostaining with many colors.^1^

The selection of antibody panels is one of the most important aspects for optimal applicability of immunohistochemistry.^2^ Studies from Jensen and colleagues^60^,^61^ concluded that the selection of the antibody panel and the interpretation of the reaction patterns of each case were the most important factors for the final diagnostic outcome.^60^ This observation was fundamental because the detection sensitivity of the chosen panel evidently increases with increased practice and experience of the pathologist who indicates the method, combined with the clinical data analysis by the researchers.^39^ Prescott and colleagues^62^ attributed 42.1% of the diagnostic discrepancies in immunohistochemistry to poor antibody selection.

The knowledge of each reagents’ characteristics, especially those of antibodies, requires new titration in each new batch or clone, selecting the dilution that offers the greatest “true/background positivity” contrast.^2^,^39^

The primary antibodies can be divided into two categories: poly or monoclonal. The polyclonal group are those obtained from animal immunization (example: rabbit, goat, monkey, rat, mouse, ewe etc) and results in antibodies that are capable of recognizing many epitopes of the same antigen, generating higher detection sensitivity. The monoclonal type, however, are developed from hybrids and provide antibodies against only one antigen epitope, yielding more specific results.^1^,^28^,^63^

Regarding the validation of findings and their interpretation, it is necessary to observe the reactivity patterns of the negative and positive, internal and external controls. The external controls (histological slices of specific tissues for each antibody) must be included in each panel, prepared from the samples fixed under the same conditions as the test cases and submitted to the same stages of the reaction. Attention must also be paid to the reactivity of structures present on the slide of the case being studied that may be used as internal positive controls, such as the reactivity of vessels for vimentin, muscle and endothelial markers, or breast ducts adjacent to the neoplasm for estrogen and progesterone receptors. Similarly, structures knowingly negative for a marker offer an excellent internal negative control, since they were submitted to the same treatment as the test-tissue, for example the erythrocytes within blood vessels—a great endogenous source of peroxidase.^2^,^23^,^56^,^64^,^65^

## Interpretation of Immunohistochemistry Expression

The interpretation of immunohistochemistry expression is generally made in a qualitative and subjective manner, whereas quantification is considered of little or no importance.^66^ Frequently, a diagnostic decision is based on cellular presence or absence of a particular molecule.^67^

Nowadays, an increasing cause of contradictory results in the literature is the lack of a definition on what constituted a positive result. In the majority of specialized studies,^66^ a result known as “positive” refers simply to the presence of brown staining (peroxidase) in any part of the studied tissue. Some authors however, extrapolate this definition and consider it a wider concept, leading to confounding factors. An example of this dilemma is the interpretation of S-100 protein expression that, for some authors, must be nuclear and cytoplasmic, while for others the staining of only the cytoplasm is sufficient to consider the immunoexpression positive.^68^ In a similar manner, studies with the HER2 protein where positive cases can include those in which the staining was exclusively cytoplasmic.^69^–^72^ However it is known that only the cases with staining for the cellular membrane are associated to the amplification of its gene as determined by molecular methods of detection.^73^ The answer to this question is to consider as positive the slide that presents brown staining (positive) and to then analyze the expression of the target-molecule in a clinical-morphologic context. The immunoexpression in different cellular compartments or in extra-cellular matrix components of the same marker can indicate that it is performing distinct or even opposite biological functions. It is fundamental, for the correct interpretation of a immunohistochemistry expression, to know the functions and the biological phenomena in which the studied molecule is involved and, based on this knowledge, the real clinical relevance of this immunoexpression can be defined.^74^

There is a rule that tries to avoid some of these problems: when the location of a target-molecule is known, the immunoreactivity pattern must follow the micro-anatomic or subcellular (cellular compartment) distribution of the antigen.^66^ For example, a granular intra-cytoplasmatic pattern should be observed when antibodies that detect molecules contained in cytoplasmatic vesicles (examples: chromogranin, von Willebrand factor, HMB-45) are employed.^2^ In this way, when the staining of an antibody, whose function is well documented and known, does not manifest as expected many authors consider it a false-positive result.^48^ However, it is important to emphasize that the researcher can be confronted with another biological function of the studied molecule, as yet undescribed.

The validation of results in cases of ambiguity can be solved using antibodies against different epitopes of the same molecule, or by the detection of correlated antibodies (for example, synaptophysin and chromogranin are both frequently expressed in neuroendocrine tumors).^2^,^66^

However, when the nature of an antigen or its function has not yet been totally elucidated, determining if the positivity of a given immunohistochemistry expression is relevant could prove hard.^66^ The possibility exists of a false-positive result, but also that the cell in question plays distinct biological roles depending on the cellular compartment where the immunostaining is present.

The interpretation of immunostaining depends on the quantity of antigen present in the tissue and according to some authors,^66^,^75^ on the determination of cut off values between what must be considered as positive and negative results, although it is important to emphasize that this quantifying methodology is not adequate. These values are often arbitrarily determined, not obtained by other laboratories and its intra-laboratory reproducibility has frequently not yet been tested.^66^ Among all these reasons, certainly the inter-laboratory reproducibility of the results of immunohistochemistry reactions is one of the most difficult challenges faced.^66^,^75^

In order to minimize these discrepancies, Seidal and colleagues^66^ suggest that more accurate quantifying methods should be adopted and studies encouraged that are dedicated to developing and refining them.

## Quantification of Immunohistochemistry Expression

Soon after the introduction of immunohistochemistry as a routine technique in pathology laboratories, efforts were made in order to try quantify protein expression using immunohistochemistry.^76^–^78^ Many studies have demonstrated that there is a correlation between the results obtained from the immunohistochemistry quantification and the tissue concentration of the antigen in question.^64^,^79^,^80^

The biological colorations (which includes those performed with aniline, hematoxylin and/or eosin for example) are usually difficult to control in terms of staining intensity. This makes the comparison from cell to cell difficult as well as from slide to slide (between different tissues and between slides prepared on different days). This difficulty tends to decrease with the introduction of automatic techniques of coloration.^81^

The reagents employed in the immunohistochemistry technique present the potential to give true quantitative results. Most researchers, however, do not consider this possibility because they often do not observe the fact that this technique is no more than an immunological test carried out *in situ* or in histological slices. The obtained staining by the immunohistochemistry technique is analogous to the results obtained in an ELISA test (*enzyme-linked immunosorbent assay*), a method recognized worldwide as truly quantitative. Exactly the same reagents that are applied in a serum test of ELISA can be employed for immunohistochemistry reactions in histological slices of specimens in paraffin blocks. Nevertheless, it is curious that the application of the same immunological principles and reagents are accepted as truly quantitative in the ELISA test, but when applied in histological slices (immunohistochemistry) are called merely “staining”. 81 The statistically significant correlation between the result from the quantification of the immunohistochemistry reaction and protein levels have been demonstrated through various measurement methods,^82^ including *Western blotting*^83^–^87^ and immunoenzymatic methods.^88^–^91^

As the need for an accurate immunostaining measurement is rising, quantitative biochemical methods of tissue detection are being progressively substituted by immunohistochemistry.^66^ Some question whether this precision is in fact achievable, or even necessary. However, advances in molecular biology and the emergence of new treatments for cancer will certainly increase the demand for precise results of a series of new molecules or target-genes, as a patient selection method for a given treatment. An example that already exists is the use of trastuzumabe in patients with breast carcinoma that presents 3+ or greater immunostaining for the HER2 oncogene.^92^ Therefore, studies will be developed in a progressively higher number of tissues, because the immunohistochemistry will likely be the chosen tool in the detection of these molecules.

Many semi-quantitative measurement methods of immunohistochemistry reactions based on visual scores have been proposed in an attempt to improve this quantification.^93^,^94^ However, image analysis assisted by computer is proving superior compared to visual estimates in the establishment of quantitative results of immunohistochemistry reactions.^95^

### Semi-quantitative analysis

As seen previously, the tissue expression of biomarkers employed in the immunohistochemistry technique can occur in different cellular compartments and even in extra-cellular matrix constituents.^66^,^96^ The evaluation of this reactivity may vary from essentially positive or negative to immunostaining intensity and/or extension,^96^ which constitutes an attempt towards immunohistochemistry technique quantification, frequently denoted in the literature as a “semiquantitative method”.

When the intensity is the evaluation focus, the inclusion of reaction controls containing different levels of staining are required for comparing criteria. Subjective scores, such as those that categorize the reaction in groups of null, weak, moderate and intense immunostaining, depend very much on the researcher’s experience and are therefore unsuitable.^66^,^96^

To estimate the extension of immunostaining can also vary from a meticulous counting to a “glance” over the slides.^96^ The evaluation of the percentage of labeled cells trough the categorization of scores of the obtained percentage are less precise. Examples of the employment of this methodology includes the count of micro-metastases in bone marrow samples or the measurement of peritumoral blood vessels. In these examples minimal variances of immunoreactivity intensity, attributed to the staining method or to the fixation procedure, have little impact on the quantification itself. A similar attempt of quantification can be exemplified by the estimate of the proliferation index through immunostaining by Ki67 (MIB-1), or by factors related to the cellular cycle such as P53 and P21, in which a simple count leads to the quantity of normal and neoplastic cells. Such methods demonstrate low reproducibility and consistency of results in terms of cut off values with relevant sensitivity.^66^

Score systems were introduced to clinical practice in an attempt to overcome variances, particularly for markers that aim to select patients for specific treatments. It is important to emphasize that all scores, including those mainly used in daily practice, have demonstrated statistically significant relevance with regards to clinical variables when used by experienced researchers in the area, although they are laborious and fatiguing.

### Computer-assisted quantitative analysis

The computer-assisted image analysis has been in use since the 1980s,^97^,^98^ without a well defined historical sequence, and has proved superior to the semiquantitative method, especially in terms of its quantification accuracy in many kinds of markers,^82^,^89^,^99^–^123^ representing the solution for the reproducibility and applicability of the semi-quantitative score systems, because it yields itself to the desired quantitative result.^83^ Comparative studies demonstrate that, in controlled circumstances, the system of image analysis was superior to the manual methods when performed by many observers.^117^,^124^–^126^

### Perspectives

The future perspectives point to new discoveries that should make the immunostaining methods simpler. An already available example was the introduction of the reactions performed in a single stage (EPOS).^127^ This involves an inert polymer in which many molecules from the primary antibody and peroxidase are chemically connected, consequently decreasing the number of incubation stages, and is currently commercially available. Other important achievements were the development of semi-automatic machines especially devised for the immunohistochemistry technique^128^ and the *microarray* technology that will be fundamental in the selection of proteins implicated in diagnosis, prognosis and therapeutic decisions of many diseases.^129^ Despite its high cost, this type of technology could be fundamental to pathology laboratories in which the diagnostic routine is very extensive. In addition it could also be of great value in the standardization of the employed technique and the reproducibility of the results.^1^

Without doubt, the development of quantification methods for the immunohistochemistry technique, mainly those which are computer-assisted, have increased not only the accuracy in the detection of markers, but also the reliability of their results. Most larger laboratories, until recently, were those which held this technology compared to small laboratories and academic centers, largely due to economic reasons. 81,130 However, with the recent spread, practicality, reproducibility and reliability of obtained results along with falling costs of systems of computerassisted image analysis is changing this panorama. At present, immunohistochemistry quantification is widely employed in many areas, not only in pathology, but also in various medical areas with particular impact in the clinical daily practice.

Future developments of the immunohistochemistry technique and its expression quantification should not be disseminated in two languages—that of the pathologist and that of the clinician or surgeon. The scientific, diagnostic and prognostic applications of this methodology must be explored in a bid to benefit of patient. In order to achieve this goal the collaboration and pooling of knowledge between these two valuable medical areas is vital.

## References

[1] Brandtzaeg P The increasing power of immunohistochemistry and immunocytochemistry J Immunol Methods 1998 216 49 67 9760215 10.1016/s0022-1759(98)00070-2

[2] Alves VAF Bacchi CE Vassallo J Manual de Imuno-histoquímica São Paulo Sociedade Brasileira de Patologia 1999

[3] Haines DM West KH Immunohistochemistry: forging the links between immunology and pathology Vet Immunol Immunopathol 2005 108 151 6 16143404 10.1016/j.vetimm.2005.08.007

[4] Marrack J Nature of antibodies Nature 1934 133 292 3

[5] Coons AH Creech HJ Jones RN Immunological properties of an antibody containing a fluorescence group Proc Soc Exp Biol Med 1941 47 200 2

[6] Avrameas S Uriel J Method of antigen and antibody labelling with enzymes and its immunodiffusion application C R Acad Sci Hebd Seances Acad Sci D 1966 262 2543 5 4958634

[7] Nakane PK Pierce GB Jr Enzyme-labeled antibodies: preparation and application for the localization of antigens J Histochem Cytochem 1966 14 929 31 17121392 10.1177/14.12.929

[8] Nakane PK Simultaneous localization of multiple tissue antigens using the peroxidase-labeled antibody method: a study on pituitary glands of the rat J Histochem Cytochem 1968 16 557 60 5717715 10.1177/16.9.557

[9] Sternberger LA Hardy PH Jr Cuculis JJ The unlabeled antibody enzyme method of immunohistochemistry: preparation and properties of soluble antigen-antibody complex (horseradish peroxidase-antihorseradish peroxidase) and its use in identification of spirochetes J Histochem Cytochem 1970 18 315 33 4192899 10.1177/18.5.315

[10] Mason DY Sammons R Alkaline phosphatase and peroxidase for double immunoenzymatic labelling of cellular constituents J Clin Pathol 1978 31 454 60 77279 10.1136/jcp.31.5.454 PMC1145303

[11] Mason DY Sammons R Rapid preparation of peroxidase: anti-peroxidase complexes for immunocytochemical use J Immunol Methods 1978 20 317 24 418119 10.1016/0022-1759(78)90267-3

[12] Cordell JL Falini B Erber WN Immunoenzymatic labeling of monoclonal antibodies using immune complexes of alkaline phosphatase and monoclonal anti-alkaline phosphatase (APAAP complexes) J Histochem Cytochem 1984 32 219 29 6198355 10.1177/32.2.6198355

[13] Singer SJ Preparation of an electron-dense antibody conjugate Nature 1959 183 1523 4 13666799 10.1038/1831523a0

[14] Sternberger LA Electron microscopic immunocytochemistry: a review J Histochem Cytochem 1967 15 139 59 5340570 10.1177/15.3.139

[15] Faulk WP Taylor GM An immunocolloid method for the electron microscope Immunochemistry 1971 8 1081 3 4110101 10.1016/0019-2791(71)90496-4

[16] Roth J. Bullock GR Petrusz P Techniques in Immunocytochemistry Academic Press 1982 107 20

[17] Huang SN Minassian H More JD Application of immunofluorescent staining on paraffin sections improved by trypsin digestion Lab Invest 1976 35 383 90 62099

[18] Hsu SM Raine L Protein A, avidin, and biotin in immunohistochemistry J Histochem Cytochem 1981 29 1349 53 6172466 10.1177/29.11.6172466

[19] Hsu SM Raine L Fanger H A comparative study of the peroxidase-antiperoxidase method and an avidin-biotin complex method for studying polypeptide hormones with radioimmunoassay antibodies Am J Clin Pathol1981a 75 734 8 6165237 10.1093/ajcp/75.5.734

[20] Hsu SM Raine L Fanger H The use of antiavidin antibody and avidin-biotin-peroxidase complex in immunoperoxidase technics Am J Clin Pathol1981b 75 816 21 6167159 10.1093/ajcp/75.6.816

[21] Hsu SM Raine L Fanger H Use of avidin-biotin-peroxidase complex (ABC) in immunoperoxidase techniques: a comparison between ABC and unlabeled antibody (PAP) procedures J Histochem Cytochem1981c 29 577 80 6166661 10.1177/29.4.6166661

[22] Leong AS Wright J The contribution of immunohistochemical staining in tumour diagnosis Histopathology 1987 11 1295 305 3326815 10.1111/j.1365-2559.1987.tb01874.x

[23] Taylor CR Cote RJ Immunomicroscopy: a diagnostic tool for surgical pathologist Major Problems in Pathology Nova Iorque WB Sauders 1994

[24] Rosai J Special techniques in surgical pathology Ackerman’s Surgical Pathology Nova Iorque Mosby-Year Book 1996 29 62

[25] Swanson PE HIERanarchy: the state of the art in immunohistochemistry Am J Clin Pathol 1997 107 139 40 9024061 10.1093/ajcp/107.2.139

[26] Bodey B The significance of immunohistochemistry in the diagnosis and therapy of neoplasms Expert Opin Biol Ther 2002 2 371 93 11955276 10.1517/14712598.2.4.371

[27] Werner B Campos AC Nadji M Uso prático da imuno-histoquímica em patologia cirúrgica J Bras Patol Med Lab 2005 41 353 64

[28] Nadji M Immunoperoxidase techniques. I. Facts and artifacts Am J Dermatopathol 1986 8 32 6 3518521 10.1097/00000372-198602000-00006

[29] Rickert RR Maliniak RM Intralaboratory quality assurance of immunohistochemical procedures. Recommended practices for daily application Arch Pathol Lab Med 1989 113 673 9 2471487

[30] Schmitt FC Utilidade dos métodos imuno-histoquímicos para o diagnóstico anatomopatológico Rev Hosp Clin Fac Med Sao Paulo 1991 46 26 30 1843001

[31] Torres LFB Noronha L Telles J A importância da imuno-histoquímica no diagnóstico anatomopatológico em hospital geral: análise de 885 casos J Bras Patol Med Lab 1995 31

[32] Torres LFB A contribuição da imuno-histoquímica em patologia cirúrgica: experiência de 10 anos Rev Med Paraná 1998 56 31 8

[33] Weiss LM Chang KL Practical applications of immunohistochemistry. Short course handout United States and Canadian Academy of Pathology Annual Meeting 1999

[34] Raab SS The cost-effectiveness of immunohistochemistry Arch Pathol Lab Med 2000 124 1185 91 10923081 10.5858/2000-124-1185-TCEOI

[35] Taylor CR The total test approach to standardization of immunohistochemistry Arch Pathol Lab Med 2000 124 945 51 10888767 10.5858/2000-124-0945-TTTATS

[36] Werner M Chott A Fabiano A Effect of formalin tissue fixation and processing on immunohistochemistry Am J Surg Pathol 2000 24 1016 9 10895825 10.1097/00000478-200007000-00014

[37] Hsi ED A practical approach for evaluating new antibodies in the clinical immunohistochemistry laboratory Arch Pathol Lab Med 2001 125 289 94 11175655 10.5858/2001-125-0289-APAFEN

[38] Jaffer S Bleiweiss IJ Beyond hematoxylin and eosin—the role of immunohistochemistry in surgical pathology Cancer Invest 2004 22 445 65 15493365 10.1081/cnv-200034896

[39] Yaziji H Barry T Diagnostic Immunohistochemistry: what can go wrong Adv Anat Pathol 2006 13 238 46 16998317 10.1097/01.pap.0000213041.39070.2f

[40] Lewis SM Quality assurance programmes in the United Kingdom Ann Ist Super Sanita 1995 31 53 9 8546375

[41] External evaluation of technical quality of immunohistochemistry. Results of a preliminary multicenter study. Immunohistochemistry Commission of the French Association of Quality Assurance in Pathology and Cytology (AFAQAP-IHC) Ann Pathol 1997 17 129 33 9221006

[42] Frisman DM “Immunoquerry.”Retrieved 11 de janeiro de 2007.

[43] Shi SR Liu C Taylor CR Standardization of Immunohistochemistry for Formalin-fixed, Paraffin-embedded Tissue Sections Based on the Antigen Retrieval Technique: From Experiments to Hypothesis J Histochem Cytochem 2006 39 741 8 16982846 10.1369/jhc.6P7080.2006

[44] Jacobs TW Prioleau JE Stillman IE Loss of tumor marker-immunostaining intensity on stored paraffin slides of breast cancer J Natl Cancer Inst 1996 88 1054 9 8683636 10.1093/jnci/88.15.1054

[45] Manne U Myers RB Srivastava S Re: loss of tumor marker-immunostaining intensity on stored paraffin slides of breast cancer J Natl Cancer Inst 1997 89 585 6 9106651 10.1093/jnci/89.8.585

[46] Wick MR Algorithmic immunohistologic analysis of undifferentiated neoplasms United States an Canadian Academy of Pathology Annual Meeting 1995

[47] Wasielewski R Komminoth P Werner M Influence of fixation, antibody clones, and signal amplification on steroid receptor analysis Breast J 1998 44 33 40

[48] Alves VA Wakamatsu A Kanamura CT The importance of fixation in immunohistochemistry: distribution of vimentin and cytokeratins in samples fixed in alcohol and formol Rev Hosp Clin Fac Med Sao Paulo 1992 47 19 24 1284893

[49] Balaton AL Coindre JM Collin F Recommendations for the immunohistochemical evaluation of hormone receptors on paraffin sections of breast cancer. Study Group on Hormone Receptors using Immunohistochemistry FNCLCC/AFAQAP. National Federation of Centres to Combat Cancer/French Association for Quality Assurance in Pathology Ann Pathol 1996 16 144 8 8767687

[50] Shi SR Key ME Kalra KL Antigen retrieval in formalin-fixed, paraffinembedded tissues: an enhancement method for immunohistochemical staining based on microwave oven heating of tissue sections J Histochem Cytochem 1991 39 741 8 1709656 10.1177/39.6.1709656

[51] Cattoretti G Becker MH Key G Monoclonal antibodies against recombinant parts of the Ki-67 antigen (MIB 1 and MIB 3) detect proliferating cells in microwave-processed formalin-fixed paraffin sections J Pathol 1992 168 357 63 1484317 10.1002/path.1711680404

[52] Leong AS Applied immunohistochemistry for the surgical pathologist London Edward Arnold Publishers 1993

[53] Giorno R A comparison of two immunoperoxidase staining methods based on the avidin-biotin interaction Diagn Immunol 1984 2 161 6 6388981

[54] Shi ZR Itzkowitz SH Kim YS A comparison of three immunoperoxidase techniques for antigen detection in colorectal carcinoma tissues J Histochem Cytochem 1988 36 317 22 3278057 10.1177/36.3.3278057

[55] Elias JM Gaborc D A comparison of the peroxidase-anti-peroxidase (PAP), avidin-biotin complex (ABC) and labeled avidin-biotin (LAB) methods for detection of glucagon in paraffin embedded human pancreas Am J Clin Pathol 1989 92 62 2546420 10.1093/ajcp/92.1.62

[56] Elias JM Gown AM Nakamura RM Quality control in immunohistochemistry. Report of a workshop sponsored by the Biological Stain Commission Am J Clin Pathol 1989 92 836 43 2480062 10.1093/ajcp/92.6.836

[57] Pastore JN Clampett C Miller JM A rapid immunoenzyme immunolabeling technique using EPOS reagens J Histotech 1995 18 35 40

[58] Bobrow MN Harris TD Shaughnessy KJ Catalyzed reporter deposition, a novel method of signal amplification. Application to immunoassays J Immunol Methods 1989 125 279 85 2558138 10.1016/0022-1759(89)90104-x

[59] Bobrow MN Litt GJ Shaughnessy KJ The use of catalyzed reporter deposition as a means of signal amplification in a variety of formats J Immunol Methods 1992 150 145 9 1613251 10.1016/0022-1759(92)90073-3

[60] Jensen HE Salonen J Ekfors TO The use of immunohistochemistry to improve sensitivity and specificity in the diagnosis of systemic mycoses in patients with haematological malignancies J Pathol 1997 181 100 5 9072010 10.1002/(SICI)1096-9896(199701)181:1<100::AID-PATH100>3.0.CO;2-O

[61] Jensen ML Nielsen O Johansen P Immunohistochemistry in tumor diagnosis. External quality assessment of 13 Departments of Pathology in Western Denmark Appl Immunohistochem 1997 5 35 44

[62] Prescott RJ Wells S Bisset DL Audit of tumour histopathology reviewed by a regional oncology centre J Clin Pathol 1995 48 245 9 7730487 10.1136/jcp.48.3.245 PMC502460

[63] Lipman NS Jackson LR Trudel LJ Monoclonal versus polyclonal antibodies: distinguishing characteristics, applications, and information resources Ilar J 2005 46 258 68 15953833 10.1093/ilar.46.3.258

[64] Elias JM Immunohistopathology: a practical approach to diagnosis Chicago ASCP Press 1990

[65] Taylor CR An exaltation of experts: concerted efforts in the standardization of immunohistochemistry Hum Pathol 1994 25 2 11 7508882 10.1016/0046-8177(94)90164-3

[66] Seidal T Balaton AJ Battifora H Interpretation and quantification of immunostains Am J Surg Pathol 2001 25 1204 7 11688582 10.1097/00000478-200109000-00013

[67] Collins RD Is clonality equivalent to malignancy: specifically, is immunoglobulin gene rearrangement diagnostic of malignant lymphoma Hum Pathol 1997 28 757 9 9224740 10.1016/s0046-8177(97)90145-3

[68] Radotra BD Joshi K Kak VK Choroid plexus tumours—an immunohistochemical analysis with review of literature Indian J Pathol Microbiol 1994 37 9 19 7522222

[69] Wrba F Gullick WJ Fertl H Immunohistochemical detection of the c-erbB-2 proto-oncogene product in normal, benign and malignant cartilage tissues Histopathology 1989 15 71 6 2670729 10.1111/j.1365-2559.1989.tb03042.x

[70] Hall PA Hughes CM Staddon SL The c-erbB-2 proto-oncogene in human pancreatic cancer J Pathol 1990 161 195 200 2202801 10.1002/path.1711610305

[71] Zhau HE Zhang X von Eschenbach AC Amplification and expression of the c-erb B-2/neu proto-oncogene in human bladder cancer Mol Carcinog 1990 3 254 7 1978777 10.1002/mc.2940030503

[72] Field JK Spandidos DA Yiagnisis M C-erbB-2 expression in squamous cell carcinoma of the head and neck Anticancer Res 1992 12 613 9 1377893

[73] Hicks DG Tubbs RR Assessment of the HER2 status in breast cancer by fluorescence *in situ* hybridization: a technical review with interpretive guidelines Hum Pathol 2005 36 250 61 15791569 10.1016/j.humpath.2004.11.010

[74] Stabenow E Tavares MR Ab’Saber AM Angiogenesis as an indicator of metastatic potential in papillary thyroid carcinoma CLINICS 2005 60 233 40 15962085 10.1590/s1807-59322005000300009

[75] Alves VAF Leandro LO Vassallo J Controle de qualidade interlaboratorial em imuno-histoquímica: citoceratinas e receptor de estrógeno como modelos J Bras Patol Med Lab 2004 40 175 83

[76] Polak JM Pearse AG Joffe S Quantification of secretion release by acid, using immunocytochemistry and radioimmunoassay Experientia 1975 31 462 4 1091497 10.1007/BF02026380

[77] Polak JM Pearse AG Van Mourik M Circadian rhythms of the endocrine pancreas. A quantitative biochemical and immumocytochemical study Acta Hepatogastroenterol (Stuttg) 1975 22 118 22 1093349

[78] Fritz P Hones J Lutz D Quantitative immunohistochemistry: standardization and possible application in research and surgical pathology Acta Histochem Suppl 1989 37 213 9 2505317

[79] True LD Quantitative immunohistochemistry: a new tool for surgical pathology Am J Clin Pathol 1988 90 324 5 3046322 10.1093/ajcp/90.3.324

[80] Becker RL Jr Standardization and quality control of quantitative microscopy in pathology J Cell Biochem 1993 Supp l 17G 199 204 10.1002/jcb.240531137 8007698

[81] Taylor CR Levenson RM Quantification of immunohistochemistry—issues concerning methods, utility and semiquantitative assessment II Histopathology 2006 49 411 24 16978205 10.1111/j.1365-2559.2006.02513.x

[82] Brey EM Lalani Z Johnston C Automated selection of DAB-labeled tissue for immunohistochemical quantification J Histochem Cytochem 2003 51 575 84 12704205 10.1177/002215540305100503

[83] Venter DJ Tuzi NL Kumar S Overexpression of the c-erbB-2 oncoprotein in human breast carcinomas: immunohistological assessment correlates with gene amplification Lancet 1987 2 69 72 2885574 10.1016/s0140-6736(87)92736-x

[84] Podhajsky RJ Bidanset DJ Caterson B A quantitative immunohistochemical study of the cellular response to crush injury in optic nerve Exp Neurol 1997 143 153 61 9000454 10.1006/exnr.1996.6354

[85] Dias P Chen B Dilday B Strong immunostaining for myogenin in rhabdomyosarcoma is significantly associated with tumors of the alveolar subclass Am J Pathol 2000 156 399 408 10666368 10.1016/S0002-9440(10)64743-8 PMC1850049

[86] Rieux C Carney R Lupi D Analysis of immunohistochemical label of Fos protein in the suprachiasmatic nucleus: comparison of different methods of quantification J Biol Rhythms 2002 17 121 36 12002159 10.1177/074873002129002410

[87] de Matos LL Machado LN Sugiyama MM Tecnologia aplicada na detecção de marcadores tumorais Arq Med ABC 2005 30 19 25

[88] Aasmundstad TA Haugen OA Johannesen E Oestrogen receptor analysis: correlation between enzyme immunoassay and immunohistochemical methods J Clin Pathol 1992 45 125 9 1371776 10.1136/jcp.45.2.125 PMC495650

[89] Lehr HA Mankoff DA Corwin D Application of photoshop-based image analysis to quantification of hormone receptor expression in breast cancer J Histochem Cytochem 1997 45 1559 65 9358857 10.1177/002215549704501112

[90] Bhatnagar J Tewari HB Bhatnagar M Comparison of carcinoembryonic antigen in tissue and serum with grade and stage of colon cancer Anticancer Res 1999 19 2181 7 10472328

[91] Simone NL Remaley AT Charboneau L Sensitive immunoassay of tissue cell proteins procured by laser capture microdissection Am J Pathol 2000 156 445 52 10666374 10.1016/S0002-9440(10)64749-9 PMC1850045

[92] Baselga J Perez EA Pienkowski T Adjuvant trastuzumab: a milestone in the treatment of HER-2-positive early breast cancer Oncologist 2006 11 Suppl 1 4 12 16971734 10.1634/theoncologist.11-90001-4

[93] Heyderman E Warren PJ Haines AM Immunocytochemistry today—problems and practice Histopathology 1989 15 653 8 2606461 10.1111/j.1365-2559.1989.tb01635.x

[94] Biesterfeld S Veuskens U Schmitz FJ Interobserver reproducibility of immunocytochemical estrogen- and progesterone receptor status assessment in breast cancer Anticancer Res 1996 16 2497 500 8917341

[95] Press MF Pike MC Chazin VR Her-2/neu expression in node-negative breast cancer: direct tissue quantitation by computerized image analysis and association of overexpression with increased risk of recurrent disease Cancer Res 1993 53 4960 70 8104689

[96] Walker RA Quantification of immunohistochemistry—issues concerning methods, utility and semiquantitative assessment I Histopathology 2006 49 406 10 97 16978204 10.1111/j.1365-2559.2006.02514.x

[97] Schuh D Steidl R Voss K The differential diagnosis of follicular adenomas and carcinomas in fine needle biopsies of the thyroid gland by means of automatic image analysis Zentralbl Allg Pathol 1980 124 557 60 7210935

[98] Bacus S Flowers JL Press MF The evaluation of estrogen receptor in primary breast carcinoma by computer-assisted image analysis Am J Clin Pathol 1988 90 233 9 2458030 10.1093/ajcp/90.3.233

[99] Zhu QY Analysis of blood vessel invasion by cells of thyroid follicular carcinoma using image processing combined with immunohistochemistry Zhonghua Yi Xue Za Zhi 1989 69 573 5 40 2620265

[100] Holschbach A Kriete A Schaffer R Differential diagnosis of papillary carcinomas of the thyroid, using image analysis and three dimensional reconstruction from serial sections Verh Dtsch Ges Pathol 1990 74 270 4 1708600

[101] Jensen MH Davis RK Derrick L Thyroid cancer: a computer-assisted review of 5287 cases Otolaryngol Head Neck Surg 1990 102 51 65 2106118 10.1177/019459989010200109

[102] McClelland RA Finlay P Walker KJ Automated quantitation of immunocytochemically localized estrogen receptors in human breast cancer Cancer Res 1990 50 3545 50 2187598

[103] McClelland RA Wilson D Leake R A multicentre study into the reliability of steroid receptor immunocytochemical assay quantification. British Quality Control Group Eur J Cancer 1991 27 711 5 1829909 10.1016/0277-5379(91)90171-9

[104] Goto M Nagatomo Y Hasui K Chromaticity analysis of immunostained tumor specimens Pathol Res Pract 1992 188 433 7 1384012 10.1016/S0344-0338(11)80033-6

[105] Salmon I Kiss R Franc B Comparison of morphonuclear features in normal, benign and neoplastic thyroid tissue by digital cell image analysis Anal Quant Cytol Histol 1992 14 47 54 1558615

[106] Borghi H Calle A Sesboue R Light microscopical detection of inter-alpha-trypsin inhibitor and its different mRNAs in cultured hepatoma Hep G2 cells using immunocytochemical and *in situ* hybridization techniques Histochem J 1994 26 252 61 7515868

[107] Huang X Chen S Tietz EI Immunocytochemical detection of regional protein changes in rat brain sections using computer-assisted image analysis J Histochem Cytochem 1996 44 981 7 8773563 10.1177/44.9.8773563

[108] Montironi R Diamanti L Thompson D Analysis of the capillary architecture in the precursors of prostate cancer: recent findings and new concepts Eur Urol 1996 30 191 200 8875200 10.1159/000474169

[109] Ruifrok AC Quantification of immunohistochemical staining by color translation and automated thresholding Anal Quant Cytol Histol 1997 19 107 13 9113303

[110] Smejkal GB Shainoff JR Enhanced digital imaging of diaminobenzidenestained immunoblots Biotechniques 1997 22 462 9067023 10.2144/97223bm20

[111] Tseleni S Kavantzas N Yova D Findings of computerised nuclear morphometry of papillary thyroid carcinoma in correlation with known prognostic factors J Exp Clin Cancer Res 1997 16 401 6 9505213

[112] Gordower L Decaestecker C Kacem Y Galectin-3 and galectin-3-binding site expression in human adult astrocytic tumours and related angiogenesis Neuropathol Appl Neurobiol 1999 25 319 30 10476049 10.1046/j.1365-2990.1999.00192.x

[113] Kohlberger PD Breitenecker F Kaider A Modified true-color computer-assisted image analysis versus subjective scoring of estrogen receptor expression in breast cancer: a comparison Anticancer Res 1999 19 2189 93 10472329

[114] Ma W Lozanoff S A full color system for quantitative assessment of histochemical and immunohistochemical staining patterns Biotech Histochem 1999 74 1 9 10190254 10.3109/10520299909066470

[115] Vilaplana J Lavialle M A method to quantify glial fibrillary acidic protein immunoreactivity on the suprachiasmatic nucleus J Neurosci Methods 1999 88 181 7 10389664 10.1016/s0165-0270(99)00016-3

[116] Matkowskyj KA Schonfeld D Benya RV Quantitative immunohistochemistry by measuring cumulative signal strength using commercially available software photoshop and matlab J Histochem Cytochem 2000 48 303 12 10639497 10.1177/002215540004800216

[117] Lehr HA Jacobs TW Yaziji H Quantitative evaluation of HER-2/neu status in breast cancer by fluorescence *in situ* hybridization and by immunohistochemistry with image analysis Am J Clin Pathol 2001 115 814 22 11392876 10.1309/AJ84-50AK-1X1B-1Q4C

[118] Ruifrok AC Johnston DA Quantification of histochemical staining by color deconvolution Anal Quant Cytol Histol 2001 23 291 9 11531144

[119] Underwood RA Gibran NS Muffley LA Color subtractive-computer-assisted image analysis for quantification of cutaneous nerves in a diabetic mouse model J Histochem Cytochem 2001 49 1285 91 11561013 10.1177/002215540104901011

[120] Hardie DC Gregory TR Hebert PD From pixels to picograms: a beginners’ guide to genome quantification by Feulgen image analysis densitometry J Histochem Cytochem 2002 50 735 49 12019291 10.1177/002215540205000601

[121] King TW Brey EM Youssef AA Quantification of vascular density using a semiautomated technique for immunostained specimens Anal Quant Cytol Histol 2002 24 39 48 11865948

[122] McGinley JN Knott KK Thompson HJ Semi-automated method of quantifying vasculature of 1-methyl-1-nitrosourea-induced rat mammary carcinomas using immunohistochemical detection J Histochem Cytochem 2002 50 213 22 11799140 10.1177/002215540205000209

[123] de Matos LL Stabenow E Tavares MR Immunohistochemistry quantification by a digital computer-assisted method compared to semiquantitative analysis Clinics 2006 61 417 24 17072439 10.1590/s1807-59322006000500008

[124] Cross SS Observer accuracy in estimating proportions in images: implications for the semiquantitative assessment of staining reactions and a proposal for a new system J Clin Pathol 2001 54 385 90 11328839 10.1136/jcp.54.5.385 PMC1731421

[125] Umemura S Itoh J Itoh H Immunohistochemical evaluation of hormone receptors in breast cancer: which scoring system is suitable for highly sensitive procedures Appl Immunohistochem Mol Morphol 2004 12 8 13 15163012 10.1097/00129039-200403000-00002

[126] Diaz LK Sneige N Estrogen receptor analysis for breast cancer: current issues and keys to increasing testing accuracy Adv Anat Pathol 2005 12 10 9 15614160 10.1097/00125480-200501000-00003

[127] Mayer G Bendayan M Amplification methods for the immunolocalization of rare molecules in cells and tissues Prog Histochem Cytochem 2001 36 3 85 11194866 10.1016/s0079-6336(01)80002-4

[128] Brigati DJ Budgeon HT Under ER Immunocytochemistry is automated: development of a robotic workstation based upon the capillary action principle J Histotechnol 1988 11 165 83

[129] Coindre JM Immunohistochemistry in the diagnosis of soft tissue tumours Histopathology 2003 43 1 16 12823707 10.1046/j.1365-2559.2003.01639.x

[130] Erler BS Chein K Marchevsky AM An image analysis workstation for the pathology laboratory Mod Pathol 1993 6 612 8 8248118

